# Ellagic Acid Inhibits *Trichophyton rubrum* Growth via Affecting Ergosterol Biosynthesis and Apoptotic Induction

**DOI:** 10.1155/2020/7305818

**Published:** 2020-10-27

**Authors:** Zhi-Jian Li, Amima Abula, Abudumijiti Abulizi, Chun Wang, Qin Dou, Youlidouzi Maimaiti, Abudoujilili Abudouaini, Shi-Xia Huo, Silafu Aibai

**Affiliations:** ^1^Department of Toxicology Laboratory, Xinjiang Institute of Traditional Uyghur Medicine, Urumqi, Xinjiang 830049, China; ^2^State Key Laboratory of Natural and Biomimetic Drugs, Department of Pharmacology, School of Basic Medical Sciences, Peking University, Beijing 100191, China; ^3^College of Pharmacy, Xinjiang Medical University, Urumqi, Xinjiang 830011, China

## Abstract

**Background:**

*Trichophyton rubrum*, among other dermatophytes, is a major causative agent for superficial dermatomycoses like onychomycosis and tinea pedis, especially among pediatric and geriatric populations. Ellagic acid (EA) and shikonin (SK) have been reported to have many bioactivities, including antifungal activity. However, the mechanism of EA and SK on *Trichophyton rubrum* has not yet been reported.

**Objectives:**

The purposes of this study were to evaluate the antifungal activities of EA and SK against *Trichophyton rubrum* and to illuminate the underlying action mechanisms.

**Methods:**

The effect of EA (64, 128, and 256 *μ*g/mL) and SK (8, 4, and 2 *μ*g/mL) on *Trichophyton rubrum* was investigated with different doses via detecting cell viability, ultrastructure with using a scanning electron microscope (SEM), cell apoptosis and necrosis by using the flow cytometry instrument technique (FCIT), and the ergosterol biosynthesis pathway-related fungal cell membrane key gene expressions in vitro.

**Results:**

SEM detection revealed that the *T. rubrum* cell surface was shrivelled, folded, and showed deformation and expansion, visible surface peeling, and broken hyphae, and cell contents overflowed after being treated with EA and SK; the cell apoptosis rate was significantly increased in dose-dependent manner after *T. rubrum* was treated with EA and SK; the qPCR results showed that mRNA expression of MEP4 and SUB1 was downregulated in EA- and SK-treated groups.

**Conclusions:**

Overall, our results revealed the underlying antifungal mechanism of EA and SK, which may be related to the destruction of the fungal cell membrane and inhibition of C14 demethylase and the catalytic rate of squalene cyclooxidase in the ergosterol biosynthesis pathway via downregulation of MEP4 and SUB1, suggesting that EA and SK have the potential to be developed further as a natural antifungal agent for clinical use.

## 1. Introduction

Fungal infections of the hair, skin, and nails are a common global problem, and approximately 20–25% of the worldwide population are affected by superficial fungal infections [1]. Such infections are primarily caused by *Trichophyton rubrum* and other dermatophytes. The main etiological agent of dermatophytosis is the anthropophilic and cosmopolitan fungus *Trichophyton rubrum*, which accounts for 69.5% of all dermatophytic infections [2]. Despite the achievements in antifungal study, infectious diseases caused by a great variety of clinically significant species of fungi remain a major global health concern due to the development of antifungal drug resistance [3, 4]. Synthetic preservatives and fungicides, including amphotericin B, ketoconazole, fluconazole, terbinafine, and flucytosine, have been used for decades to control fungal spoilage. However, the indiscriminate use of these substances has caused health problems for humans and animals due to their carcinogenicity, teratogenicity, and acute toxicity [5]. Hence, investigating for efficient, nontoxic, or low-toxic alternatives with potent broad-spectrum antifungal activity is an important goal in new drug research [4, 6].

**Figure 1:**
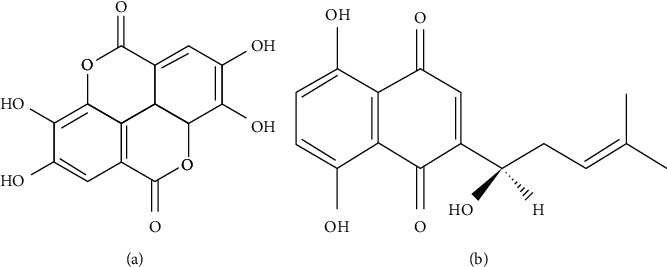
Molecular structure of (a) ellagic acid and (b) shikonin.

*Euphorbia humifusa* Willd (Euphorbiaceae) is used by traditional Uyghur medical practitioners to treat dermatomycoses including tinea pedis, tinea manuum, tinea versicolor, and tinea capitis in Xinjiang, China [7]. EA (Figure 1(a)) is the major component of *Euphorbia humifusa* [8]. It was reported that EA has a great variety of pharmacological activities, including antioxidant, antibacterial, anti-inflammatory, metabolic syndrome, neuroprotective, hepatoprotective, and skin protection activity [9]. Researchers found that ellagic acid inhibited *Candida parapsilosis* and *C. neoformans* more effectively than *Candida krusei* [10]. A study showed that EA has protective effect against fungal infection and is a promising tool for the treatment of *Candida albicans* [11]. Researchers reported antifungal activity of EA, the major compound of ethanolic and aqueous extracts of *R. echinus*, against multiresistant strains of fungi (*C. albicans, C. krusei,* and *C. tropicalis*) [12]. Our research team found that ellagic acid has a broad spectrum of antifungal activity in vitro. The best antifungal activity was observed against *Trichophyton rubrum*, and a significant decrease was demonstrated in the content of ergosterol in the *Trichophyton rubrum* membrane by ellagic acid at all concentrations, suggesting that the ergosterol biosynthesis pathway was inhibited by ellagic acid [13].

SK (Figure 1(b)), a major constituent of the red pigment extracts from the roots of the plant *Lithospermum erythrorhizon* Sieb. et Zucc (LE) in traditional Chinese medicine, has antioxidant, anti-inflammatory, anticancer, neuroprotective, hepatoprotective, antimicrobial, and antifungal activity [14].

Papageorgiou et al. reviewed conflicting studies regarding the activity of shikonin and deoxyshikonin against *Candida albicans* [15].

Shikonin showed fungicidal activity against *Candida krusei, Saccharomyces cerevisiae,* and *C. glabrata*. Deoxyshikonin showed against *C. krusei* and *S. cerevisiae* [16].

Researchers demonstrated that shikonin is active against *C. albicans*, and mitochondrial aerobic respiration and endogenous reactive oxygen species were identified as being involved in the antifungal activity of SK [17]. But, effect of ellagic acid and shikonin against *Trichophyton rubrum* on its ultrastructure changes, apoptosis, necrosis, and ergosterol biosynthesis-related target gene expressions has not been reported yet.

In the present work, we described the antifungal activity and mechanism of EA against *Trichophyton rubrum* via detecting cell viability, ultrastructure with using SEM, cell apoptosis and necrosis by using FCIT, and the ergosterol biosynthesis pathway-related fungal cell membrane key gene expressions in vitro.

## 2. Materials and Methods

### 2.1. Strain and Cultivation

The strain used in this study was the *T. rubrum* strain and was purchased from the American Type Culture Collection (ATCC). The strain was stored at −80°C and refreshed and incubated in the RPMI-1640 medium at 28°C for 7 d till the strains reached the exponential growth phase. The revived *T. rubrum* cells were pooled by centrifugation at 10000 r·min^−1^ for 15 min. After washing twice by sterile phosphate-buffered saline (PBS, Aobo Biotechnology Co., Ltd., Shanghai, China), the fungal cells were resuspended in the RPMI-1640 medium (Gibco, United States) and adjusted to a defined cell density using the blood cell counting plate prior to following tests.

### 2.2. Chemicals

Ellagic acid (EA) and shikonin (SK) (purity > 98%) were purchased from Nanjing Jingzhu Bio-Technology Co., Ltd. (Nanjing, Jiangsu, China). Terbinafine hydrochloride (TERB) was purchased from Sigma-Aldrich Co. LLC. (Shanghai, China). EA and TERB were dissolved in 100% dimethyl sulfoxide (DMSO; Scientific Research), following the protocols of the National Committee for Clinical Laboratory Standards. TERB was used as positive controls. Stock solutions of 1 mg/ml were prepared and stored at −20°C until use and diluted with the morpholinepropanesulfonic acid- (MOPS-) buffered RPMI 1640 test medium to yield twice the final strength required for the test. EA, SK, and TERB final drug concentrations ranged from 32 to 128 *μ*g/ml, 4 *μ*g/mL, and 0.04 *μ*g/ml with double gradient dilution, respectively.

### 2.3. Inoculum Suspension Preparation

Inoculum suspensions of filamentous fungi were prepared by the method of NCCLS M38-A. In brief, fungi were grown on PDA at 25 to 28°C until day 7 for *Trichophyton rubrum*. The colonies were suspended in approximately 1 ml of sterile 0.85% saline by gently probing them with the tip of a Pasteur pipette. The resulting mixture of conidia, sporangiospores, and hyphae fragments was transferred to a sterile tube. Heavier particles were allowed to settle for 3 to 5 min, and the upper homogenous suspensions were collected and mixed with a vortex mixer for 15 s. The densities of the cell suspensions of *Trichophyton rubrum* were adjusted using a spectrophotometer (Cintra 20, GBC, Australia) at a wavelength of 520 nm to a transmittance level of 70% to 72%. The suspensions of filamentous fungi were diluted 1 : 50 in the RPMI 1640 medium, corresponding to twice the density required for testing (approximately 0.4 × 10^4^ to 5 × 10^4^ cfu/ml). Inoculum quantification was made by plating 0.01 ml of a 1 : 100 dilution of the adjusted inoculum on Sabouraud dextrose agar (SDA, Difco) to determine the viable number of cfu/ml. The plates were incubated at 28–30°C and observed daily for the presence of fungal colonies. The double-strength conidial and sporangiospore inoculum suspension was approximately 5 × 10^4^ cfu/ml.

### 2.4. Cell Viability Assay

Aliquots of 100 *μ*l of the twofold drug dilutions were inoculated into the wells with a multichannel pipette. The microplates were stored at −70°C prior to use. Each microplate was inoculated with 100 *μ*l of the diluted inoculum suspensions. Cells were included for each assay, and tests were performed in duplicate. The microplates of *T. rubrum* were incubated at 28°C and read visually after 7 days of incubation. Cell viability was determined using the CCK-8 assay (Dojindo Laboratories, Kumamoto, Japan). In the cell viability assay, 10 *μ*l CCK-8 solution was added into each well of the plate. The cell viabilities were analyzed at 450 nm wavelengths after 4 h of incubation. The experiments were repeated three times.

### 2.5. Scanning Electron Microscopy (SEM)

After exposure of the *T. rubrum* strain to the compounds EA and SK for 7 d, *T. rubrum* cells were fixed at 4°C with 3% glutaraldehyde in sodium cacodylate buffer 0.1 M, pH 6.8 (Sigma-Aldrich, Shanghai, China) for 2 h and washed 3 times with PBS. Then, the samples were postfixed in 1% osmium tetroxide (OsO_4_) (Sigma-Aldrich, Beijing, China) in cacodylate buffer (Sigma-Aldrich, Shanghai, China) for 2 h at 4°C. After this step, washing and dehydration were performed in a series of increasing ethanol for 20 min each (30%, 50%, 70%, 90%, and 100%), dehydrated with acetone 3 times for 20 min each, and then immigrated to a 4°C refrigerator for 30 min. The yeasts were subjected to critical point using liquid CO_2_, coated with colloidal gold, and examined under SEM (1430VP, Germany).

### 2.6. Apoptosis Assay

Quantitative analysis of fungal cell apoptosis and necrosis was performed by flow cytometry. The *T. rubrum* strain was incubated with various concentrations of EA (256, 128, and 64 *μ*g/mL), shikonin (4 *μ*g/mL), and TERB (0.08 *μ*g/mL), incubated at 28°C for 7 d, harvested by centrifugation at 1000 r·min^−1^ for 5 min, then washed with cold PBS, and incubated with Annexin V-PE and PI following the manufacturer's protocol, using 5 *μ*l Annexin V and 10 *μ*l PI at 37°C for 20 min. The percentages of necrosis and apoptosis were analyzed using a BD Accuri C6 flow cytometer (EPICS ALTRA, Beckman, USA).

### 2.7. Quantitative RT-PCR

Quantitative RT-PCR was used to evaluate the transcription level of *MEP4* and *SUB1* genes involved in the ergosterol biosynthesis pathway during exposure of *T. rubrum* to EA and SK. Total RNA was extracted from the mycelia grown for 7 d in the presence of EA (256, 128, and 64 *μ*g/mL), SK (4 *μ*g/mL), and TERB (0.08 *μ*g/mL), and cells that were not treated with drugs served as the control samples by using the Trizol reagent according to the manufacturer's instructions. In brief, a 1 : 5 volume of chloroform was added, and the tube was vortexed and subjected to centrifugation at 12,000 g for 15 min. The aqueous phase was isolated, and the total RNA was precipitated by cold absolute ethanol. After centrifugation and washing, the total RNA was finally eluted in 20 *μ*L of RNase- and DNase-free water. The quantity was characterized using a UV spectrophotometer (NanoDrop8000, Thermo Scientific, USA). The isolated RNA has a 260/280 ratio of 1.8–2.2. Total RNA from per sample was reverse transcribed (RT) to cDNA by using PrimeScriptTM Reverse Transcriptase (Takara, Osaka, Japan) before amplifying by the SYBR Premix Ex Taq TM kit (Takara Biotech). Thermal cycling was performed in the ABI PRISM ®7500 Sequence Detection System (Applied Biosystems, Foster City, CA, USA). The gene-specific primers are shown in Table 1. The change of mRNA quantity for the genes relative to the control was calculated using the 2^−ΔΔ*CT*^ method with beta-tubulin as internal normalization. The PCR reaction was determined by melting temperature analysis.

### 2.8. Statistical Analysis

Results were expressed as mean ± SD. The overall significance of the results was determined by one-way analysis of variance using the SPSS version 16.0 statistical software package. Probability values (*P*) less than 0.05 were considered statistically significant.

The authors confirm that the ethical policies of the journal, as noted on the journal's author guidelines page, have been adhered to. No ethical approval was required as the research in this article was related to microorganisms.

## 3. Results

### 3.1. Effect of EA on Cell Viability of *T. rubrum*

**Figure 2:**
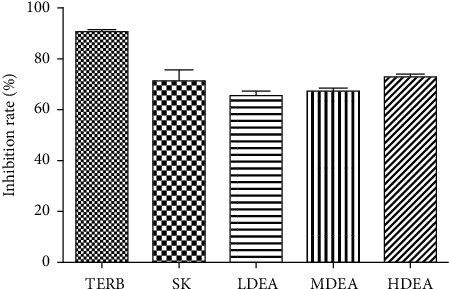
Effect of ellagic acid and shikonin on the inhibition rate (%) of *Trichophyton rubrum*. Terbinafine hydrochloride (TERB) 0.08 *μ*g/mL, shikonin (SK) 4 *μ*g/mL, and ellagic acid: low dose 32.0 *μ*g/mL (LDEA), medium dose 64.0 *μ*g/mL (MDEA), and high dose 128 *μ*g/mL (HDEA). Data are presented as mean ± SD, *n* = 3.

The current experiment was conducted to test the effect of EA and SK on cell survival of *T. rubrum*. The inhibition ratio of EA-treated *T. rubrum* cells at 7 d with the concentrations of 64, 128, and 256 *μ*g/mL of EA reached the peak value 65.34%, 67.40%, and 72.88% and the TERB- and SK-treated *T. rubrum* groups reached 90.63% and 71.17%, respectively, indicating its potential cytotoxic activity, as shown in Figure 2.

### 3.2. Effect of EA on Mycelial Morphology of *T. rubrum*

**Figure 3:**
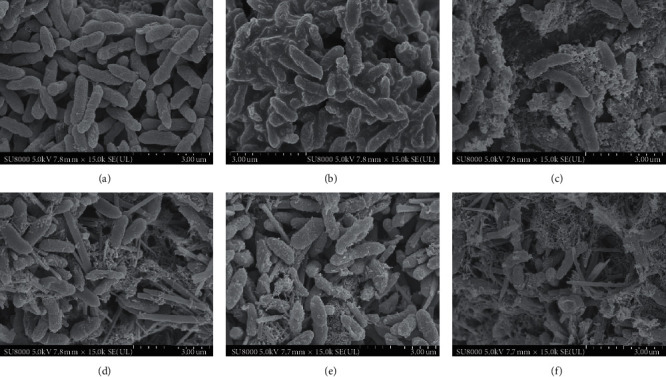
Effect of ellagic acid and shikonin on mycelial morphology of *Trichophyton rubrum*. (a) Control group, (b) terbinafine hydrochloride (TERB, 0.08 *μ*g/mL), (c) shikonin (SK, 4 *μ*g/mL), and ellagic acid: (d) low dose 32.0 *μ*g/mL (LDEA), (e) medium dose 64.0 *μ*g/mL (MDEA), and (f) high dose 128 *μ*g/mL (HDEA).

Morphological observations by SEM exhibited that treatment with EA showed significant morphological changes in size, shape, hyphae, and biomass when compared with the untreated control (Figure 3).  Figure 3(a) shows that untreated *T. rubrum* demonstrated smooth surface, complete cell structure, uniform cytoplasm, branching, and good growth. The changes were observed in the *T. rubrum* surface after treatment with EA, SK, and TERB. The hyphae morphology of *T. rubrum* was basically normal, but only a few hyphae appeared rough and with swollen surface in the LDEA group (Figure 3(d)). Obvious changes, mycelium dry deformation, many damage, and fracture were found in mycelium morphology when treated with MDEA (Figure 3(e)). Meanwhile, mycelia generally showed obvious deformation and expansion, visible surface peeling, mycelia rupture, mushy, cell content overflow in the HDEA group (Figure 3(f)). Furthermore, overflow of cytoplasmic contents, massive concentration, incomplete cell wall, formation of a large number of holes, and holes with curved mycelium penetration were detected in the mycelia of *T. rubrum* treated with shikonin (Figure 3(c)), and mycelia showed overflow of cytoplasmic contents, massive concentration, incomplete cell wall, short, hollow, irregular, and even disintegrated at different levels in *T. rubrum* treated with TERB groups (Figure 3(b)).

### 3.3. Effect of EA on Apoptosis of *T. rubrum*

**Figure 4:**
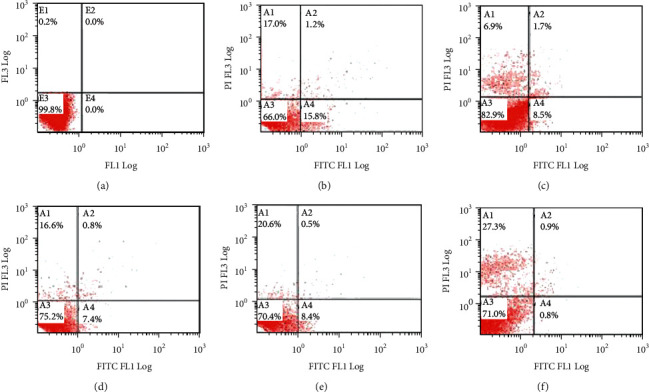
Effect of EA on apoptosis of *Trichophyton rubrum*. (a) Control group, (b) terbinafine hydrochloride (TERB, 0.08 *μ*g/mL), (c) shikonin (SK, 4 *μ*g/mL), and ellagic acid: (d) low dose 32.0 *μ*g/mL (LDEA), (e) medium dose 64.0 *μ*g/mL (MDEA), and (f) high dose 128 *μ*g/mL (HDEA).

The apoptosis level of each group was detected by Annexin V/PI double staining (Figure 4). The results showed that after *T. rubrum* cells were treated with different concentrations of TERB, SK, and EA for 7 d, the number of normal cells in A3 decreased while apoptotic cells in A1, 2, and 4 increased gradually, and the number of apoptotic cells in the control group (Figure 4(a)), TERB (Figure 4(b)), SK (Figure 4(c)), LDEA (Figure 4(d)), MDEA (Figure 4(e)), and HDEA (Figure 4(f)) was 0.2%, 44%, 17.1%, 24.8%, 29.6%, and 29.0%, respectively.

### 3.4. Effect of EA and SK on the Related Gene Expression of Biosynthesis of *T. rubrum*

**Figure 5:**
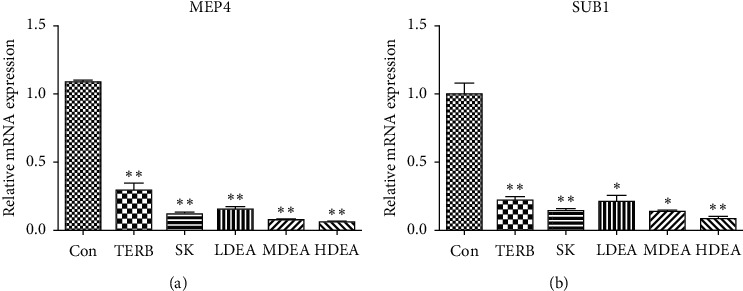
Effect of EA on the related gene expression of biosynthesis of *Trichophyton rubrum*. Control group (Con), terbinafine hydrochloride (TERB) 0.08 *μ*g/mL, shikonin (SK) 4 *μ*g/mL, and ellagic acid: low dose 32.0 *μ*g/mL (LDEA), medium dose 64.0 *μ*g/mL (MDEA), and high dose 128 *μ*g/mL (HDEA). (a) MEP4 and (b) SUB1. Data are presented as mean ± SD, *n* = 3. ^*∗*^*P* < 0.05; ^*∗∗*^*P* < 0.01 versus the untreated control group.

The RT-PCR assay was performed (Figure 5) for evaluating the gene expression levels of MEP4 and SUB1, and results showed that the mRNA expression of MEP4 and SUB1 was remarkably downregulated in all drug-treated groups in dose-dependent manner (*P* < 0.05 or *P* < 0.01).

## 4. Discussion

The frequency of life-threatening infections induced by pathogenic fungal species has augmented worldwide. These fungal species are majorly responsible for mortality and morbidity in immunocompromised patients. In spite of the severity and the high incidence of fungal infections, treatments are still insufficient and limited [18]. Human pathogenic fungal infections are more ubiquitous in both developing and developed nations [19]. According to the World Health Organization (WHO), more than 25% of the global populations are infected with various dermatophytes [19]. The profound dermatophytes have impacted patients' quality of life due to the severe pain and physical deformation caused by the infection. The treatment of fungal infections requires comprehensive treatment requirements and high treatment costs and shows severe side effects (e.g., nephrotoxicity, hepatotoxicity, and neurotoxicity) [20–22]. Furthermore, due to the very high rate of recurrence of superficial fungal infections and the increasing antifungal-resistance problems, therefore, new alternative fungicidal drugs that are safe and more effective are very much needed [23–25].

In this background, the antifungal activity of EA and SK against *T. rubrum*, one of the major human pathogenic dermatophytes, particularly in immunocompromised patients, including those undergoing bone marrow transplantation or solid organ, those with diabetes mellitus, hematological malignancy, and human immunodeficiency disorder, and those using long-term steroid therapy, was evaluated with the major clinically used drug, terbinafine. The results show that EA and SK are able to exert an efficient dose-dependent inhibitory activity on *T. rubrum* in vitro.

Although a few researchers observed ultrastructural changes of *Trichophyton rubrum* after itraconazole therapy and terbinafine therapy observed using SEM [26], the structural changes of *T. rubrum* after EA and SK treatment in vitro have not been reported previously. On the basis of the published study on the effect of terbinafine on the morphology of *Trichophyton rubrum*, in our research, the morphological observations by SEM exhibited that treatment with EA and SK showed significant morphological changes, including hyphae appearing with rough and swollen surface, mycelium dry deformation and expansion, many fracture, visible surface peeling, mycelia rupture, mushy, and cell content overflow. The majority of *T. rubrum* hyphae were dry, shrivelled, curved, and folded (Figure 3) and in accordance with the previous report [26]. The shrinkage, flattening, bending, and twisting of the hyphae with wrinkled surface after application for 7 days were also identical to the morphological changes in hyphae cultured with luliconazole [27]. Our findings are in accordance with the previous studies demonstrated.

Ergosterol is the unique lipid of the fungal cell membrane and is an important structural component of the fungal cell membrane. By binding with phospholipids, it can stabilize the membrane structure and regulate the fluidity of the fungal cell membrane. It also plays an important role in ensuring the integrity of the membrane structure, influencing the activity of membrane-bound enzymes, cell viability, and material transport. Ergosterol deficiency will cause fungal cell membrane abnormalities and even cell rupture. When SK and EA acted on *Trichophyton rubrum*, the mycelial structure was destroyed and there were many ruptures and loss of cell contents in the body of the fungus.

Subsequently, we tested in flow cytometry to evaluate the necrosis and apoptosis on *T. rubrum* interfered with target drugs. The loss of the plasma membrane asymmetry is one of the morphological alterations in early apoptosis [28]. PS, a phospholipid that exists in the inner leaflet of the plasma membrane under normal conditions, is externalized from the inner to the outer leaflet of the membrane in apoptotic cells. Therefore, exposed PS can bind Annexin V, a Ca^2+^-dependent phospholipid-binding protein, at a high affinity [29]. As shown in Figure 4, all three antifungal drug treatments could induce cell apoptosis and necrosis.

Due to the lack of studies on the mechanism of action of antifungal agents [30, 31], the determination of apoptosis has recently been a new and important approach in antifungal therapy [32]. Therefore, illustrating the phenomenon of apoptosis in fungi by activating the fungal cells to suicide is beneficial for new antifungal drug discovery strategies [33]. On this basis, the flow cytometric test was used on *T. rubrum* for defining apoptotic properties of EA and SK. While TERB showed significant effect on inducing cell apoptosis that 15.8% of the cell underwent early apoptosis, 1.2% of the cell underwent late apoptosis, and 17% necrosis, the percentages of apoptotic cells reached 1.7% and 10.2% and necrotic cells reached 27.3% and 6.9% in the presence of HDEA and SK, respectively, and necrotic cells of EA-treated groups increased in dose-dependent manner (Figure 4). These results suggest that EA and SK exerted potential antifungal activity against *T. rubrum* via programmed cell death (apoptosis) and directly killing the cells (necrosis).

EA interferes with the synthesis of ergosterol, an important component of the fungal cell membrane. Changes in the ergosterol biosynthetic pathway will also inhibit fungal growth [34]. Our research team previously found that *Euphorbia humifusa* Willd, Uyghur traditional medicine, was used to treat fungal diseases, and it can decrease the membrane CYP51 enzyme activity, gene expression of MEP and SUB, and fungal cell membrane ergosterol biosynthesis [35]. EA, a major compound of *Euphorbia humifusa* Willd, inhibited ergosterol biosynthesis and reduced the activity of sterol 14*α*-demethylase P450 (CYP51) in the *Trichophyton rubrum* membrane [35].

Terbinafine, the positive control in the in vitro experiment, is widely used clinically for treating dermatophytosis, has a primary fungicidal action, and is the most potent agent against a great variety of pathogenic fungi [36]. And, it acts by inhibiting enzymes involved in ergosterol biosynthesis [37]. In this study, we found that mRNA expression of MEP4 and SUB1 was remarkably downregulated in EA- and SK-treated groups in dose-dependent manner. The effects of EA and SK were similar to those of terbinafine.

Ergosterol is a vital component of the fungal cell, but little is known about the genetics and biochemistry of the ergosterol biosynthesis pathway, and only 20 genes involved in the biosynthesis of ergosterol in *T. rubrum* have been sequenced [38]. Resistance to antifungal agents was due to the accumulation of sterol intermediates, which is consistent with the inactivation of lanosterol demethylase, and to the increased expression of several ergosterol biosynthesis genes [39].

The secreted proteases of *T. rubrum* are considered to be the most important virulence factors in dermatophytes. In particular, members of the two endoprotease gene families of SUB and MEP have been studied [40, 41]. A seven-member subtilisin gene (SUB) family, encoding serine proteases, has been identified in *Microsporum canis*, as well as in *Trichophyton rubrum* and *T. mentagrophytes* [40, 42]. *MEP4* was identified as remarkably upregulated genes by subtractive suppression hybridization after incubation of *T. rubrum* with keratin for 72 h [43]. *SUB1* and *MEP4* genes were expressed at high levels during growth in the nail-containing medium [44]. Our research team found that *Euphorbia humifusa* Willd downregulated the mRNA expression of MEP and SUB protease in *T. rubrum*, suggesting that it can exert antifungal activity via inhibiting infection process and affecting nutrition obtaining of *T. rubrum* [35]. Our results in this research are in accordance with other researchers' work.

## 5. Conclusion

In summary, to our knowledge, this is the first study using SEM, Annexin V/PI double staining, and qPCR to analyze the changes in apoptosis, morphology, and the gene expression profile of *T. rubrum* in response to EA and SK. Antifungal mechanism of EA and SK maybe related to apoptosis, necrosis, and the destruction of the fungal cell membrane and the catalytic rate of squalene cyclooxidase in the ergosterol biosynthesis pathway via downregulation of MEP4, SUB1, and the activity of CYP51, suggesting that EA and SK have the potential to be developed further as a natural antifungal agent for clinical use.

## Figures and Tables

**Table 1:** Primer sequences of the genes selected for RT-PCR.

Gene		Primer	Sequence
*SUB1*		Forward	5′-CGGTAGGGTTCTCCTGAGCA-3′
		Reverse	5′-GTTGAACAACACGGCTGCAT-3′

*MEP4*		Forward	5′-CACCTTCCCTGGCTCAAAAC-3′
		Reverse	5′-CGGTAGGGTTCTCCTGAGCA-3′

*Beta-tubulin*		Forward	5′-AACATGATGGCTGCCACTGA-3′
		Reverse	5′-AAGATGGCAGAGCAGGTAAGGT-3′

## Data Availability

The data used to support the findings of this study are available from the corresponding author upon request.
